# Minoxidil, Platelet-Rich Plasma (PRP), or Combined Minoxidil and PRP for Androgenetic Alopecia in Men: A Cost-Effectiveness Markov Decision Analysis of Prospective Studies

**DOI:** 10.7759/cureus.20839

**Published:** 2021-12-30

**Authors:** Kevin M Klifto, Sammy Othman, Stephen J Kovach

**Affiliations:** 1 Plastic and Reconstructive Surgery, University of Missouri School of Medicine, Columbia, USA; 2 Plastic Surgery, University of Pennsylvania Perelman School of Medicine, Philadelphia, USA; 3 Plastic and Reconstructive Surgery, Northwell Health, New Hyde Park, USA

**Keywords:** cost-effectiveness analysis, platelet-rich plasma/prp, markov decision process, treatment options, quality of life (qol), minoxidil, male sex, cost analyses, health and social care, androgenetic alopecia

## Abstract

Background

Androgenetic alopecia (AGA) is the most common cause of hair loss in men. In this study, we evaluated the cost-effectiveness of minoxidil monotherapy, minoxidil and platelet-rich plasma (PRP) combined therapy, and PRP monotherapy for the long-term treatment of early-onset AGA Hamilton-Norwood stages I-V in men.

Methodology

Markov modeling was performed to analyze the base-case parameters from 18 level I/II studies. The model base-case assumes a healthy 25-year-old man presenting to a dermatologist or plastic surgeon’s office as a new patient for the evaluation and treatment of AGA Hamilton-Norwood stages I-V (non-severe AGA in men). Simulations began at an age of 25 years and ran over 35 years. Analyses were conducted from healthcare and societal perspectives. Outcomes included incremental cost-effectiveness ratios (ICER) and net monetary benefits (NMB). Willingness-to-pay (WTP) thresholds were set at $50,000 and $100,000. Deterministic and probabilistic sensitivity analyses were performed to evaluate uncertainty over 10,000 simulations.

Results

From a healthcare perspective, compared to minoxidil monotherapy, the ICER for minoxidil+PRP was $52,036/quality-adjusted-life-year (QALY) and the ICER for PRP monotherapy was $439,303/QALY. The NMB of minoxidil monotherapy was $914,887, minoxidil+PRP was $914,350, and PRP monotherapy was $904,572 at a WTP threshold of $50,000. When the WTP threshold was increased to $100,000, the NMB of minoxidil+PRP was $1,843,908, minoxidil monotherapy was $1,831,237, and PRP monotherapy was $1,822,246. Societal trends were similar.

Conclusions

Minoxidil 5% topical twice-daily monotherapy provided cost-effective treatment for men with AGA Hamilton-Norwood stages I-V at a WTP threshold of $50,000, whereas combining minoxidil 5% with PRP provided cost-effective treatment at a WTP threshold of $100,000.

Level of evidence: Level II.

## Introduction

Androgenetic alopecia (AGA) is the most common cause of hair loss in men, affecting 30-50% of men by the age of 50 years [1]. AGA in men or male pattern baldness occurs in a highly reproducible pattern, preferentially affecting the temples, vertex, and mid frontal scalp [1]. It is characterized by the miniaturization of hair follicles, shortening of the anagen (growth) phase, and increases in the percentage of telogen (resting) hair follicles, producing microscopic hairs. Although the morbidity of AGA is primarily psychological, hair loss may increase the risk of scalp skin cancers [1,2]. Consequently, patients with AGA may seek treatment from dermatologists or plastic surgeons.

Food and Drug Administration (FDA)-approved treatments for AGA in men include pharmacologic interventions minoxidil and finasteride [1]. Minoxidil is available as an over-the-counter generic topical formulation, whereas finasteride is an oral prescription medication. Although minoxidil is relatively affordable, it requires life-long twice-daily administration, strong patient compliance, and variable efficacy between individuals [3]. Newer therapies have provided alternatives for less compliant patients or additional therapies for those who are non-responsive to current therapies [1].

Platelet-rich plasma (PRP) has increased in popularity as a “safe and efficacious” treatment for AGA in men [4]. The literature defines PRP as a sample of autologous blood with platelet concentrations above normal physiologic levels, produced by the centrifugation of whole blood [5]. Its efficacy relates directly to regenerative properties promoted by locally released growth factors, estimated from over 30 biologically active proteins [6,7]. For hair restoration, PRP increases proliferation rates of human dermal papilla cells that regulate hair follicle growth [8]. Growth factors bind and interact with both dermal papilla cells and primitive stem cells, resulting in the activation of the proliferative phase of the hair cycle [6-9]. Promoting and maintaining the anagen phase of the hair cycle delays the catagen phase and increases hair density [6-9]. Although studies have provided evidence through quantitative measurements such as hair counts, immunohistochemistry, and dermoscopic photomicrographs, high costs associated with PRP may outweigh its effectiveness and clinical use [10-15]. Given the psychological burden and high out-of-pocket costs associated with life-long treatments for AGA in men, it is critical to study their cost-effectiveness. Hence, the objective of this study was to evaluate the cost-effectiveness of minoxidil monotherapy, minoxidil and PRP combined therapy, and PRP monotherapy for the long-term treatment of early-onset AGA in men.

## Materials and methods

Analytic overview

Markov modeling and Monte Carlo patient simulations were used to evaluate the cost-effectiveness of three long-term treatments for early-onset AGA in men. Markov models are decision trees that simulate clinical pathways as transitions between discrete health states. These transitions follow transition probabilities. Health states have designated quality-of-life (QOL) values (utilities) and costs. A QOL value is defined as an individual’s quantitation of disease burden on life and rated on a scale of 0 (death) to 1 (perfect health) [16]. As the model progresses, QOL values accumulate in each state into quality-adjusted life-years (QALYs). Costs include direct payments from healthcare perspectives and indirect payments (lost productivity from time off work) added to direct payments from societal perspectives [17].

In this study, model outcomes were incremental cost-effectiveness ratios (ICER), calculated by dividing cost differences by QALY differences for treatments $ICER = \Delta Cost / \Delta QALY$ and net monetary benefits (NMB), evaluated against predetermined willingness-to-pay (WTP) thresholds $NMB = WTP * QALY - Cost$ [17]. The WTP threshold was defined as the maximum cost society was willing to pay for an additional QALY, set at $50,000 and $100,000 US Dollars (USD)/QALY [18]. If the ICER for the treatment fell below the WTP threshold, it was cost-effective. Treatment with the greatest NMB value was the most cost-effective. If one treatment cost less and produced more QALYs compared to another, it was termed the dominant treatment [17].

We adhered to the Consolidated Health Economic Evaluation Reporting Standards (CHEERS) guidelines. All model designs and analyses were completed in accordance with recommendations made by the Second Panel on Cost-Effectiveness in Health and Medicine using TreeAge Healthcare software (TreeAge Software, Inc., Williamstown, MA) [17].

Markov model design

**Figure 1:**
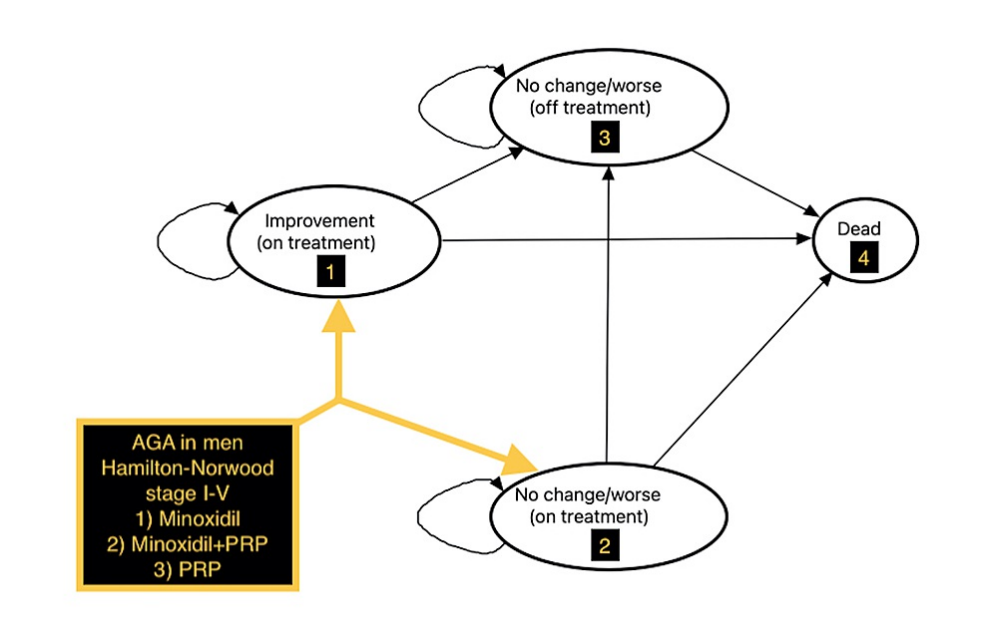
Markov model transition state diagram. Markov model representation used to evaluate the cost-effectiveness of (1) minoxidil 5% solution monotherapy versus (2) minoxidil 5% solution+PRP injections versus (3) PRP injections monotherapy for men with AGA Hamilton-Norwood scale stage I-V. The base-case patient was a 25-year-old man with AGA. Patients started the model at the time of physician evaluation. They were treated with minoxidil 5% solution twice-daily monotherapy, minoxidil 5% solution twice-daily and PRP injections every month for three months, or PRP injections as monotherapy every month for three months during the first six-month cycle. Men who received treatment demonstrated improvement (health state 1) or no change/became worse (health state 2). Over subsequent six-month, cycles patients continued treatment, discontinued treatment (health state 3), or died (health state 4) and either remained in a health state or transitioned to another health state. Straight arrows indicate transitions between health states; curved arrows indicate continuing within a health state. Thick straight arrows indicate transitions to first-cycle health states; thin straight black arrows indicate transitions to second-cycle health states. The time horizon was 35 years (ages 25-60 years). AGA: androgenetic alopecia; PRP: platelet-rich plasma

The model base-case assumed a healthy 25-year-old man who presented to a dermatologist or plastic surgeon’s office as a new patient for the evaluation and treatment of AGA Hamilton-Norwood stages I-V (non-severe AGA in men) [1]. Following an evaluation with laboratory tests to rule out secondary causes of alopecia, men were offered minoxidil 5% topical solution monotherapy, minoxidil 5% topical solution combined with PRP injections (minoxidil+PRP), or PRP injection monotherapy. Minoxidil 5% topical solution was applied to the scalp twice daily. PRP injections were administered at three office visits (0, 4, and 8 weeks), followed by every six months. Minoxidil+PRP combined therapy data were not assumed to be additive but derived from the literature [11]. Following the first six-month treatment cycle, men with AGA Hamilton-Norwood stages I-V entered two initial health states: (1) improvement (on treatment) or (2) no change/worse (on treatment). Over subsequent six-month cycles, patients remained in a health state or transitioned to another state if they continued treatment, discontinued treatment (health state 3), or died (health state 4) (Figure 1).

Patients died each year depending on age-adjusted annualized male mortality rates [19]. The model time horizon was 35 years (ages 25-60 years).

Transition probabilities

Probabilities were extrapolated from randomized controlled trials (RCTs) that evaluated the efficacy and safety of minoxidil monotherapy, minoxidil+PRP, or PRP monotherapy for men with AGA Hamilton-Norwood stages I-V [9-15,20,21]. Treatment response probabilities between health states were determined using physician assessments based on prospective clinical studies at six-month cycles. Hair pull tests, dermoscopic evaluations, and photographs were used to determine treatment responses [1]. During the first cycle, 55% of patients improved with minoxidil+PRP [12], 53% of patients improved with PRP monotherapy [9], and 51% of patients improved with minoxidil monotherapy [10,14]. Following the first six-month cycle for those who improved, 99% of patients continued minoxidil+PRP [11], 97% of patients continued PRP monotherapy [15,20], and 91% of patients continued minoxidil monotherapy [11,13,14,20,21]. Over subsequent cycles, 98% of men who improved continued treatment, while 50% of men who did not improve continued treatment (Table 1) [1,9-15,20,21].

**Table 1:** Model input parameters stratified by treatment for AGA in men. Minoxidil 5% solution was administered twice daily. *Age-adjusted annualized male mortality rates were derived from the CDC for ages 25 to 74 from years 1999 to 2019 [19]. AGA: androgenetic alopecia; PRP: platelet-rich plasma; CDC: Centers for Disease Control and Prevention; USD: US Dollar; QOL: quality of life; CMP: comprehensive metabolic panel; CBC: complete blood count; TSH: thyroid-stimulating hormone; T4: thyroxine; TIBC: total iron-binding capacity; RPR: rapid plasma regain

Treatment			Minoxidil	Minoxidil+PRP	PRP
Transition probabilities					
First 6-month cycle					
	Improvement		0.51	0.55	0.53
		Continue treatment	0.91	0.99	0.97
Subsequent six-month cycles					
	Improvement		-	-	-
		Continue treatment	0.98	0.98	0.98
	No change/worse (on treatment)		-	-	-
		Continue treatment	0.50	0.50	0.50
	No change/worse (off treatment)		0.29	0.22	0.2
	Mortality*		CDC	CDC	CDC
QOL (utilities)					
			0.89	0.92	0.89
Healthcare costs (2020 USD)					
	Initial physician visit		$150	$15	$150
	Minoxidil 5% solution (six months)		$96.60	$96.60	-
	Injection procedure CPT-0232T (PRP+lidocaine 1% per mL)		-	$717.67	$717.67
Societal costs (2020 USD)					
First six-month cycle					
	Time off from work		$166.55	$499.65	$499.65
Subsequent six-month cycles					
	Time off from work		-	$166.55	$166.55

Age-adjusted annualized male mortality rates were derived from the Centers for Disease Control and Prevention (CDC) for ages 25 to 60 years (Appendix, Table 1) [19].

Quality of life

Optimal QOL values were derived from a comprehensive review of studies by aggregating and annualizing patient-reported satisfaction scores (Table 1). The optimal QOL for men with AGA Hamilton-Norwood stages I-V was 0.85 [2,22]. We assumed the optimal QOL for men with no change/worse AGA following treatment was 0.85 (baseline) [2,22]. The QOL of AGA following improvement with minoxidil+PRP was 0.92 [11], minoxidil monotherapy was 0.89 [10,11,14,20], and PRP monotherapy was 0.89 [11].

Costs

Medicare reimbursement schedules and published studies were used to estimate costs for physician office visits, minoxidil, and PRP injections adjusted to 2020 USD (Table 1) [3,23-29]. Minoxidil treatment did not require subsequent patient-physician encounters. The average costs of six months of minoxidil were $96.60 [3]. The average costs of single PRP injections were $717.67 [24-27,29]. From a societal perspective, time off from work was calculated using US Census Bureau annual reports from full-time male employees [28]. Annual reports were converted into average half-day earnings based on a five-day workweek. Half-day earnings were lost for initial physician visits and subsequent PRP injections. Costs related to adverse effects of treatment were negligible. Adverse effects from minoxidil and PRP were minor and resolved without further interventions. Minoxidil adverse effects included pruritus of the scalp, seborrheic dermatitis, and erythema [11,13,14,20,21]. PRP adverse effects included injection site pain [9-13,15].

Discounting

All future costs and utility values were discounted at 3% to account for inflation, opportunity costs, and time preferences [17].

Sensitivity analysis

One-way and two-way deterministic sensitivity analyses were performed on all transition probabilities and QOLs varying each value from 0 to 1 to determine sensitive parameters. Costs of PRP injections were varied across ranges from $0 to $1,000. Probabilistic sensitivity analyses were performed using second-order Monte Carlo simulations to simultaneously evaluate parameters across distributions evaluated in one-way sensitivity analyses. Distributions were calculated using means and standard deviations (SD) derived from studies. Beta distributions were used for transition probabilities and QOLs, whereas gamma distributions were used for costs. A total of 10,000 iterations were performed against WTP thresholds of $50,000, $100,000, and $200,000 per QALY. Scatterplots were generated to incorporate levels of uncertainty around distributions. Cost-effectiveness acceptability curves were generated to determine the percentage of iterations in which treatments were cost-effective.

## Results

Base-case

Base-case model results were generated from both healthcare and societal perspectives, calculated over a 35-year horizon (Table 2).

**Table 2:** Cost-effectiveness rankings over 35 years of treatment at WTP threshold of $50,000 for AGA in men. AGA: androgenetic alopecia; PRP: platelet-rich plasma; USD: US dollar; QALY: quality-adjusted life-years; ICER: incremental cost-effectiveness ratio; NMB: net monetary benefits; WTP: willingness-to-pay. $ICER=\Delta Cost / \Delta QALY$; $NMB=WTP*QALY-Cost$.

Treatment		Minoxidil	Minoxidil+PRP	PRP
Healthcare perspective				
	Cost ($)	$1,463	$15,209	$13,103
	Incremental cost ($)	Dominant	$13,103	$11,640
	Effectiveness (QALY)	18.327	18.591	18.353
	Incremental effectiveness (QALY)	Dominated	0.264	0.026
	Cost-effectiveness ratio ($/QALY)	80	818	714
	ICER ($/QALY)	Dominant	$52,036	$439,303
	NMB ($)	$914,887	$914,350	$904,572
Societal perspective				
	Cost ($)	$1,629	$18,148	$15,913
	Incremental cost ($)	Dominant	$16,519	$14,284
	Effectiveness (QALY)	18.327	18.591	18.353
	Incremental effectiveness (QALY)	Dominated	0.264	0.026
	Cost-effectiveness ratio ($/QALY)	89	976	867
	ICER ($/QALY)	Dominant	$62,531	$539,073
	NMB ($)	$914,721	$911,410	$901,762

Patients who received minoxidil+PRP accumulated 18.591 QALYs, PRP monotherapy accumulated 18.353 QALYs, and minoxidil monotherapy accumulated 18.327 QALYs over 35 years. Compared to minoxidil monotherapy, minoxidil+PRP was associated with a 0.264 increase in QALYs per patient, and PRP monotherapy was associated with a 0.026 increase in QALYs per patient. The average total healthcare costs for minoxidil+PRP was $15,209, PRP monotherapy was $13,103, and minoxidil monotherapy was $1,463 over 35 years. From the healthcare perspective, minoxidil monotherapy was the most cost-effective. PRP monotherapy was the least cost-effective and dominated from the outset. Compared to minoxidil monotherapy, the ICER for minoxidil+PRP was $52,036/QALY and PRP monotherapy was $439,303/QALY (Table 2). Both minoxidil+PRP and PRP monotherapy were above the WTP threshold of $50,000, whereas minoxidil+PRP was below the WTP threshold of $100,000. The NMB of minoxidil monotherapy was $914,887, minoxidil+PRP was $914,350, and PRP monotherapy was $904,572 at a WTP threshold of $50,000. As the WTP threshold was increased to $100,000, the NMB of minoxidil+PRP was $1,843,908, minoxidil monotherapy was $1,831,237, and PRP monotherapy was $1,822,246. Societal perspectives generated similar trends (Table 2).

Deterministic sensitivity analysis

**Figure 2:**
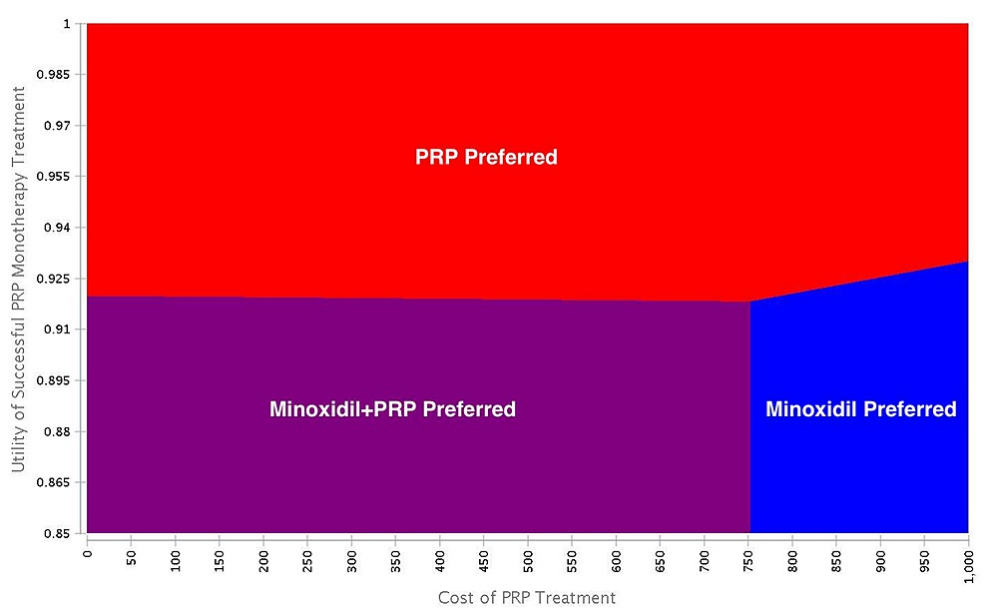
Deterministic two-way analysis from the healthcare perspective. Two-way analyses revealed if the utility scores increased above 0.92 following treatment, then PRP monotherapy would be preferred over minoxidil monotherapy at a WTP threshold of $50,000 USD. Blue = minoxidil monotherapy; purple = minoxidil+PRP; red = PRP monotherapy. PRP: platelet-rich plasma; WTP: willingness-to-pay

**Figure 3:**
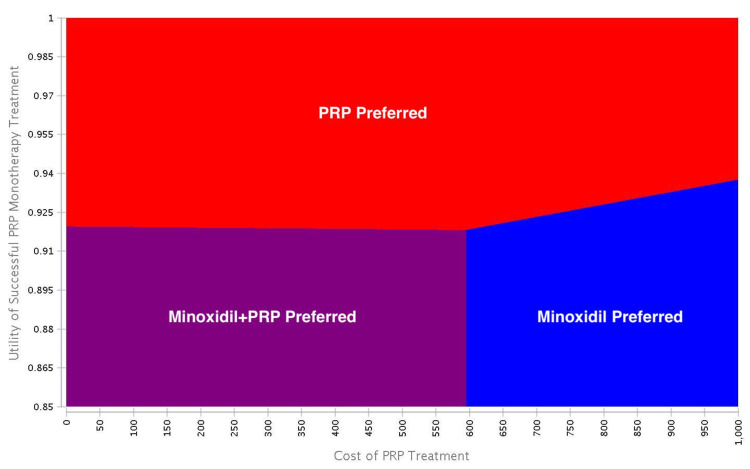
Deterministic two-way analysis from the societal perspective. Two-way analyses revealed if the utility scores increased above 0.92 following treatment, then PRP monotherapy would be preferred over minoxidil monotherapy at a WTP threshold of $50,000 USD. Blue = minoxidil monotherapy; purple = minoxidil+PRP; red = PRP monotherapy. PRP: platelet-rich plasma; WTP: willingness-to-pay

Both minoxidil and PRP monotherapy increased utility scores from 0.85 to 0.89. Utility value improvements increased by 0.04 for each treatment (Table 2). High costs associated with PRP limited cost-effectiveness. The cost of a PRP injection was $717.67 in the base-case scenario. From a healthcare perspective, sensitivity analysis revealed if the cost of a PRP injection was less than $86.71 or utility scores increased above 0.92 following treatment, then PRP monotherapy would be preferred over minoxidil monotherapy. From both healthcare and societal perspectives, two-way analyses revealed if the utility scores increased above 0.92 following treatment, then PRP monotherapy would be preferred over minoxidil monotherapy at a WTP threshold of $50,000. Trends were similar for both healthcare and societal perspectives when varying model parameters (Figures 2, 3).

Probabilistic sensitivity analysis

**Figure 4:**
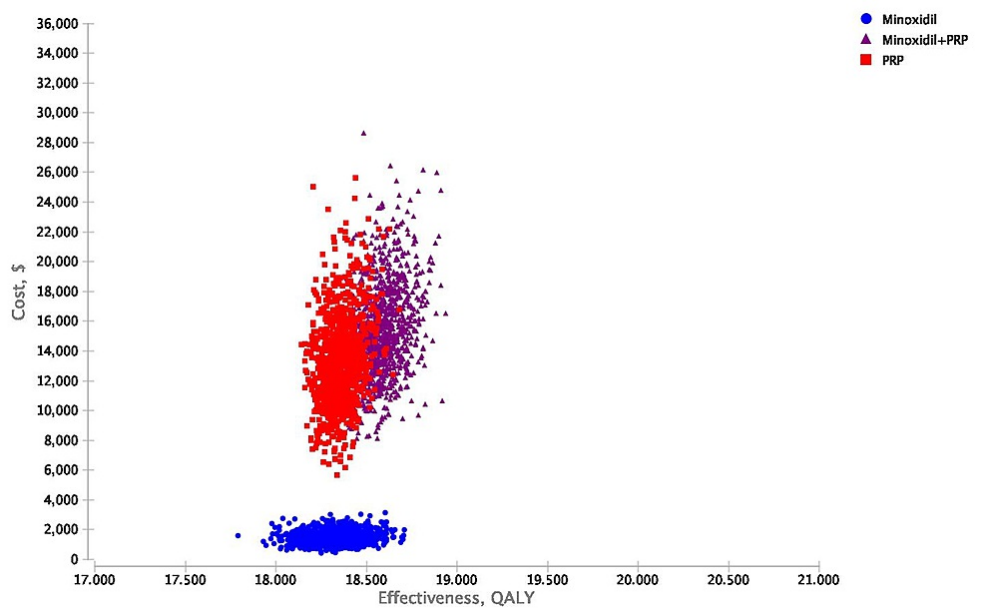
Probabilistic sensitivity analyses scatterplots illustrating cost-effectiveness results of 10,000 iterations for minoxidil monotherapy, minoxidil+PRP, and PRP monotherapy. Healthcare perspective mean ± standard deviation values. Costs: minoxidil $1,459 ± $432, PRP $13,107 ± $2,998, minoxidil+PRP $15,169 ± $3,213; QALYs: minoxidil+PRP 18.59 ± 0.11, minoxidil 18.32 ± 0.14, PRP 18.35 ± 0.08; NMB: minoxidil $914,886 ± $6,695, minoxidil+PRP $914,358 ± $5,097, PRP $904,607 ± $4,370. PRP: platelet-rich plasma; QALY: quality-adjusted life-year; NMB: net monetary benefits

**Figure 5:**
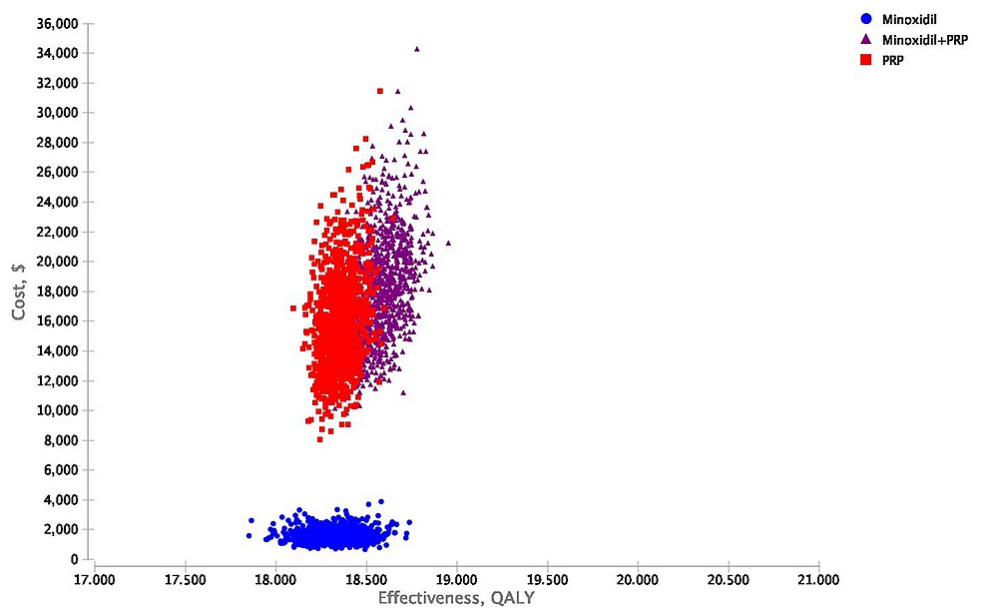
Probabilistic sensitivity analyses scatterplots illustrating cost-effectiveness results of 10,000 iterations for minoxidil monotherapy, minoxidil+PRP, and PRP monotherapy. Societal perspective mean±standard deviation values. Costs: minoxidil $1,622 ± $431, PRP $15,933 ± $3,231, minoxidil + PRP $18,190 ± $3,466; QALYs: minoxidil+PRP 18.59 ± 0.11, PRP 18.35 ± 0.08, minoxidil 18.33 ± 0.13; NMB: minoxidil $914,643 ± $6,571, minoxidil+PRP $911,400 ± $4,959, PRP $901,779 ± $4,391. PRP: platelet-rich plasma; QALY: quality-adjusted life-year; NMB: net monetary benefits

Cost-effectiveness scatterplot results of 10,000 iterations of the analysis were performed when considering uncertainty for treatment improvement probabilities, QOLs associated with a specific treatment, and costs of minoxidil and PRP from healthcare perspectives (Figures 4, 5).

**Figure 6:**
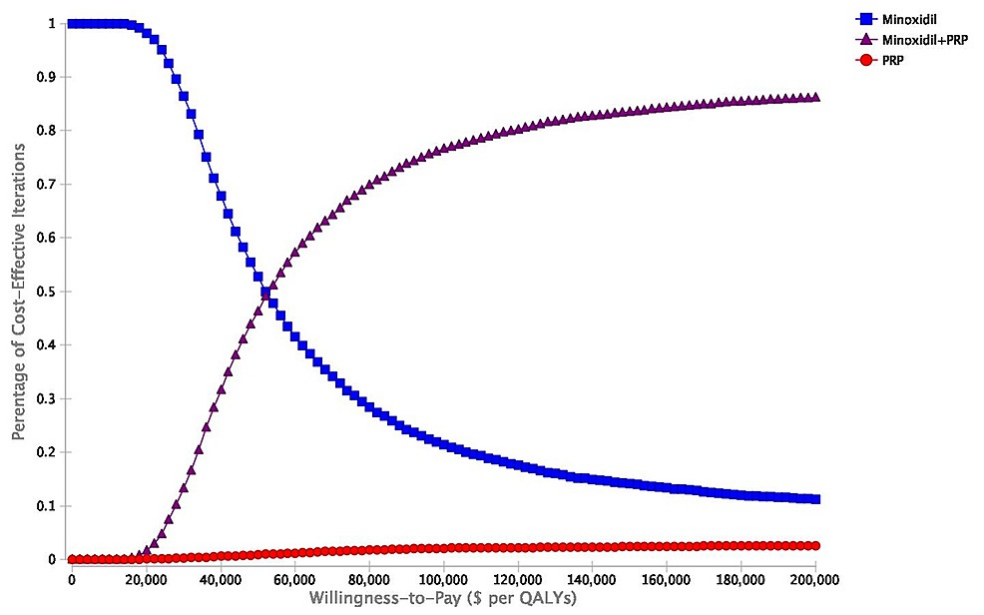
Cost-effectiveness acceptability curves illustrating the percentage of the 10,000 probabilistic sensitivity analyses iterations that demonstrated cost-effectiveness across various WTP thresholds for minoxidil monotherapy, minoxidil+PRP, and PRP monotherapy. Healthcare perspective. WTP: willingness-to-pay; PRP: platelet-rich plasma

**Figure 7:**
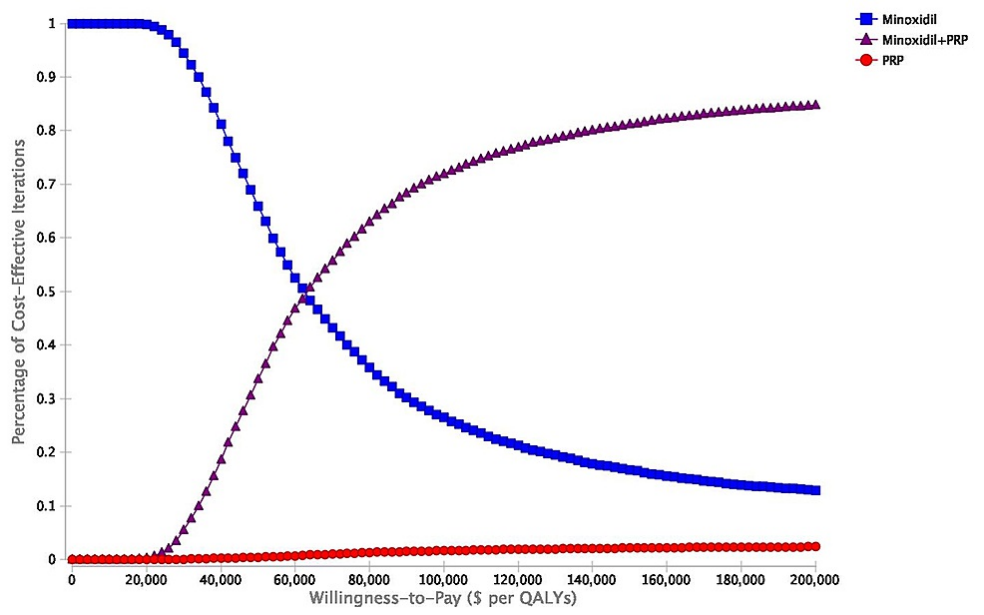
Cost-effectiveness acceptability curves illustrating the percentage of the 10,000 probabilistic sensitivity analyses iterations that demonstrated cost-effectiveness across various WTP thresholds for minoxidil monotherapy, minoxidil+PRP, and PRP monotherapy. Societal perspective. WTP: willingness-to-pay; PRP: platelet-rich plasma

Acceptability curves displayed how many of the 10,000 iterations of treatments were cost-effective across WTP thresholds from healthcare perspectives (Figures 6, 7).

**Figure 8:**
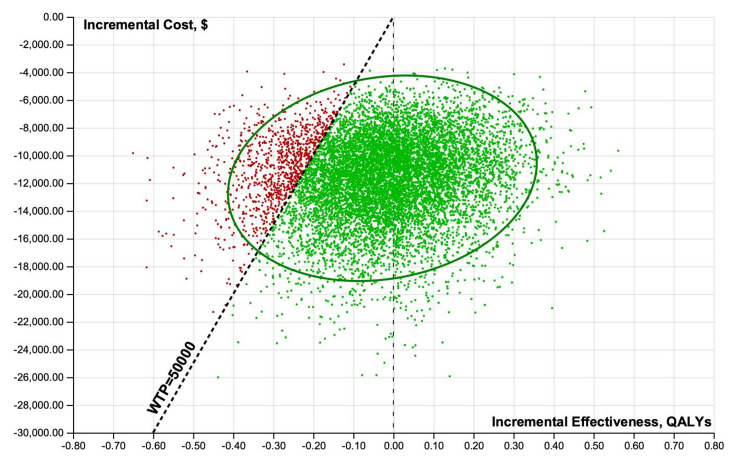
Probabilistic sensitivity analyses scatterplots illustrating incremental cost-effectiveness results of 10,000 iterations. Healthcare perspective, minoxidil versus PRP. PRP: platelet-rich plasma; QALY: quality-adjusted life-year; WTP: willingness-to-pay

**Figure 9:**
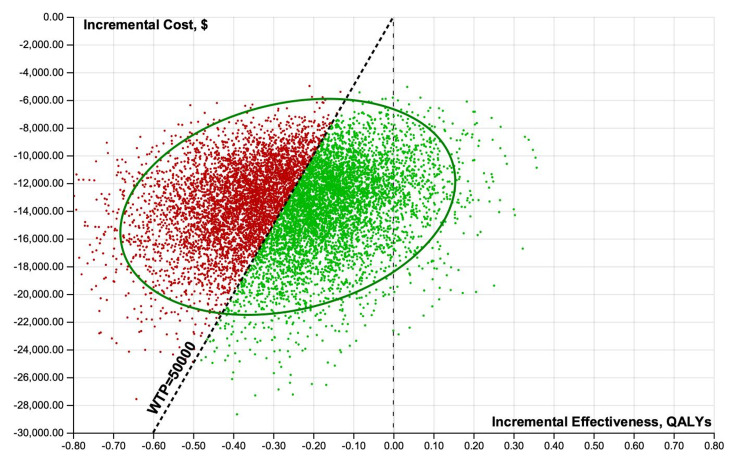
Probabilistic sensitivity analyses scatterplots illustrating incremental cost-effectiveness results of 10,000 iterations. Healthcare perspective, minoxidil versus minoxidil+PRP. PRP: platelet-rich plasma; QALY: quality-adjusted life-year; WTP: willingness-to-pay

**Figure 10:**
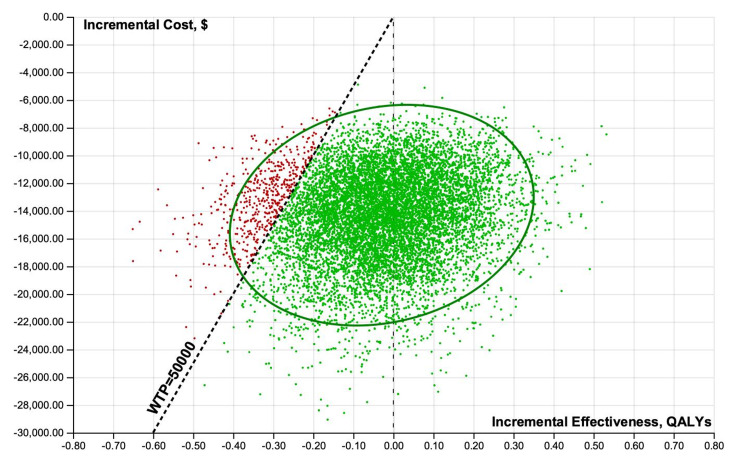
Probabilistic sensitivity analyses scatterplots illustrating incremental cost-effectiveness results of 10,000 iterations. Societal perspective, minoxidil versus PRP. PRP: platelet-rich plasma; QALY: quality-adjusted life-year; WTP: willingness-to-pay

**Figure 11:**
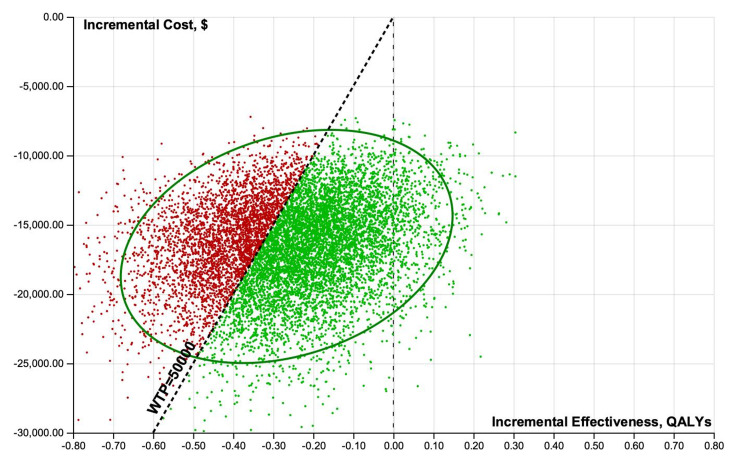
Probabilistic sensitivity analyses scatterplots illustrating incremental cost-effectiveness results of 10,000 iterations. Societal perspective, minoxidil versus minoxidil+PRP. PRP: platelet-rich plasma; QALY: quality-adjusted life-year; WTP: willingness-to-pay

Incremental cost-effectiveness scatterplot results of 10,000 iterations of the analysis were performed when considering uncertainty for treatment improvement probabilities, QOLs associated with a specific treatment, and costs of minoxidil and PRP from healthcare perspectives (Figures 8-11).

From healthcare and societal perspectives at a WTP threshold of $50,000, minoxidil was cost-effective 51-66% of the time. At a WTP threshold of $100,000 and $200,000, minoxidil+PRP was cost-effective 75-91% of the time.

## Discussion

Through a Markov cohort and Monte Carlo simulation analysis, minoxidil 5% topical solution monotherapy administered twice daily provided cost-effective treatment for men with AGA Hamilton-Norwood stages I-V at a WTP threshold of $50,000. Combining minoxidil 5% with PRP provided cost-effective treatment at WTP thresholds of $100,000 and $200,000. Both deterministic and probabilistic sensitivity analyses demonstrated that the model was reasonably robust and generalizable following variations in model parameters.

We compared PRP monotherapy and combination therapy to commonly used first-line FDA-approved medication, minoxidil [1]. Minoxidil was approved by the FDA for AGA in 1988, followed by over-the-counter availability and generic formulations in 1996 [3]. Hair growth response rates to minoxidil have been reported to range from 30% to 88% [10].

Loss of half-day salaries from work for patients receiving PRP injections contributed to differences between healthcare and societal costs. Over the 49-year time horizon, costs accumulated from two consecutive months following initial physician encounters and every six months for patients receiving PRP injections. Generic availability, over-the-counter access, and ease of administration reduce the costs of minoxidil [3]. For PRP to be more cost-effective than minoxidil at a WTP threshold of $50,000, its cost would need to be reduced to less than $86.71. Costs of PRP reported in the literature range from $4.88 to $2,301 [30]. Low costs of $4.88 reflect prices of materials required to extract and administer PRP in a setting that has a centrifuge. In the United States, PRP is billed using the temporary CPT code 0232T [23]. This CPT code is a Level III categorization with Status Code C that bundles the harvesting, preparation, and image guidance for the service [23]. The cost of $717.67 includes both the patient and physician fees [23-27,29]. In addition to the temporary CPT code, PRP is prepared inconsistently from study to study. To date, no consensus exists regarding the volume of blood required, methods of preparation, spins, centrifugations, platelet concentrations, red blood cell concentrations, white blood cell concentrations, activation substrates, anticoagulation, purity, and supernatant for PRP as a therapeutic intervention [5]. The importance of each variable has not been universally accepted. The lack of PRP consensus may limit manufacturing universally affordable standardized PRP kits. However, standardizing PRP concentrations may not be clinically practical and add unnecessary costs [5].

Limitations of this study were related to the inconsistency of PRP formulations and decision analysis methodologies. Even among the same PRP preparation kits, the product by definition is different [5]. This limitation is characteristic of all PRP studies. Model parameters were populated using available data from RCTs with men for each treatment. Minoxidil studies provided larger sample sizes with robust methodologies compared to PRP studies. Three studies reported head-to-head comparisons between minoxidil and PRP [10,11,13]. One RCT directly compared all three treatments [11]. We assumed the QOL and probabilities of responses were mutually exclusive between treatments without patients changing treatment cohorts. Patients may be more willing to try over-the-counter products prior to seeing a specialist for treatment recommendations. We assumed patients who continued therapy through more than one cycle were more likely to continue if they had improvement on treatment and 98% would continue treatment if they were compliant and motivated through subsequent cycles [1,4]. For men who had no change/worse responses, we assumed 50% would continue treatment if they were compliant and motivated through subsequent cycles [1,4]. Over subsequent cycles, men with no change/worse responses would eventually discontinue treatments as time progressed. Although time horizons extended beyond published treatment durations, assumptions were reasonable following deterministic and probabilistic sensitivity analyses. Model findings should not be applied to all patients without proper clinical context and judgment. Variations in base-case scenario parameters may potentially impact outcomes (e.g., demographics, comorbidities, Hamilton-Norwood stages VI-VII).

## Conclusions

This study evaluated the cost-effectiveness of minoxidil monotherapy, minoxidil+PRP combined therapy, and PRP monotherapy for the long-term treatment of early-onset AGA Hamilton-Norwood stages I-V in men using healthcare and societal perspectives. Minoxidil 5% topical solution monotherapy administered twice daily provided a cost-effective treatment at a WTP threshold of $50,000. For PRP to be more cost-effective than minoxidil at a WTP threshold of $50,000, its cost would need to be less than $86.71. Combining minoxidil 5% with PRP provided a cost-effective treatment at a WTP threshold of $100,000.

## Appendices

Systematic review methodologies

We used PubMed to search MEDLINE, Embase, Cochrane Library, Web of Science, and Scopus on December 29, 2020. The search terms were used to search for terms related to androgenetic alopecia (AGA) and platelet-rich plasma (PRP) and clinical studies. We limited the search to human studies with available full texts. PubMed: (“alopecia”[Mesh] OR “hair follicle”[Mesh] OR “hair”[Mesh]) AND ((“Platelet-Rich Plasma”[Mesh] OR “platelet rich plasma” OR “platelet-rich plasma” OR “PRP” [tiab]) OR (“minoxidil"[Mesh])) AND (“Clinical Studies as Topic”[Mesh] OR “Clinical Study” [Publication Type] OR “clinical study” OR “clinical studies” OR “clinical trial” OR “clinical trials” OR random* [tiab] OR control* [tiab] OR trial* [tiab]). Embase: ‘male type alopecia’ AND (‘platelet-rich plasma cell’ OR ‘minoxidil’). Web of Science: (TS=(androgenetic alopecia) AND (TS=(platelet-rich plasma) OR TS=(minoxidil) AND TS=(humans) AND LANGUAGE:(English) AND DOCUMENT TYPES:(Article) Timespan: All years. Indexes: SCI-EXPANDED, SSCI, A&HCI, CPCI-S, CPCI-SSH, BKCI-S, BKCI-SSH, ESCI, CCR-EXPANDED, IC. Cochrane Library: MeSH descriptor: [Androgenetic Alopecia] explode all trees AND (MeSH descriptor: [Platelet-Rich Plasma] explode all trees OR MeSH descriptor: [Minoxidil] explode all trees). Scopus: (TITLE-ABS-KEY (androgenetic AND alopecia) AND (TITLE-ABS-KEY (platelet-rich AND plasma) OR TITLE-ABS-KEY (minoxidil)) AND TITLE-ABS-KEY (humans) AND TITLE-ABS-KEY (clinical)) AND (LIMIT-TO (LANGUAGE, “English”)) AND (LIMIT-TO (DOCTYPE, “ar”)).

Searches from five databases resulted in 724 studies which were screened by their title and abstract. Our inclusion criteria were non-surgical clinical studies of PRP and/or minoxidil for the treatment of AGA in men with Hamilton-Norwood Scale Stages I-V, a randomized controlled trial (RCT) or prospective comparative study, and reporting in English. If there were any questions regarding the inclusion of a study, we reviewed the full text to determine eligibility. We excluded studies that did not specify PRP preparation; studied patients with Hamilton-Norwood Scale Stages VI and VII; studied other causes of alopecia; studied mixed populations of men and/or women; studied index patients out of our age range (e.g., <18 years or ≥60 years); and commentaries, reviews (except systematic reviews/meta-analyses), and methodological papers. This resulted in a total of 36 studies of interest. Full texts of these 36 studies were evaluated. To maximize the quality of literature used to derive parameters for the model, we limited our data extraction to RCTs and prospective cohort studies. This resulted in a total of 18 studies (14 level I and four level II studies) that were thoroughly reviewed to derive the data input for our model.

Appendix Table 3

**Table 3:** Annual male mortality rate adjusted for age.

Age (years)	Annual male mortality rate
25	0.0014
26	0.0014
27	0.0014
28	0.0015
29	0.0015
30	0.0015
31	0.0016
32	0.0016
33	0.0017
34	0.0017
35	0.0018
36	0.0019
37	0.0020
38	0.0021
39	0.0022
40	0.0024
41	0.0026
42	0.0027
43	0.0030
44	0.0032
45	0.0035
46	0.0038
47	0.0041
48	0.0045
49	0.0049
50	0.0053
51	0.0058
52	0.0062
53	0.0068
54	0.0073
55	0.0080
56	0.0085
57	0.0092
58	0.0100
59	0.0108
60	0.0116

Age-adjusted annualized male mortality rates were derived from the Centers for Disease Control and Prevention (CDC) for ages 25 to 60 from years 1999 to 2019.
